# The effect of missing data on design efficiency in repeated cross-sectional multi-period two-arm parallel cluster randomized trials

**DOI:** 10.3758/s13428-020-01529-7

**Published:** 2021-02-02

**Authors:** Mirjam Moerbeek

**Affiliations:** Department of Methodology and Statistics, Utrecht University, PO Box 80140, 3508 TC Utrecht, The Netherlands

**Keywords:** Cluster randomization, Dropout, Intermittently missing observations, Efficiency

## Abstract

The reduced efficiency of the cluster randomized trial design may be compensated by implementing a multi-period design. The trial then becomes longitudinal, with a risk of intermittently missing observations and dropout. This paper studies the effect of missing data on design efficiency in trials where the periods are the days of the week and clusters are followed for at least one week. The multilevel model with a decaying correlation structure is used to relate outcome to period and treatment condition. The variance of the treatment effect estimator is used to measure efficiency. When there is no data loss, efficiency increases with increasing number of subjects per day and number of weeks. Different weekly measurement schemes are used to evaluate the impact of planned missing data designs: the loss of efficiency due to measuring on fewer days is largest for few subjects per day and few weeks. Dropout is modeled by the Weibull survival function. The loss of efficiency due to dropout increases when more clusters drop out during the course of the trial, especially if the risk of dropout is largest at the beginning of the trial. The largest loss is observed for few subjects per day and a large number of weeks. An example of the effect of waiting room environments in reducing stress in dental care shows how different design options can be compared. An R Shiny app allows researchers to interactively explore various design options and to choose the best design for their trial.

**Supplementary Information:**

The online version contains supplementary material available at 10.3758/s13428-020-01529-7.

## Introduction

Over the past two decades, the cluster randomized trial (Campbell & Walters, 2014; Donner & Klar, 2000; Eldridge & Kerry, 2012; Hayes & Moulton, 2009; Murray, 1998) has become a standard design in the biomedical, health and behavioral sciences. As outcomes of subjects within the same cluster are correlated, cluster randomization has lower efficiency than individual randomization. Efficiency may be improved by increasing the sample size, but this is not always possible in practice, as the number of clusters and cluster size are often limited. Various alternative strategies have been proposed to increase efficiency, such as including covariates (Bloom, 2005; Bloom, Richburg-Hayes, & Black, 2007; De Hoop, Teerenstra, Van Gaal, Moerbeek, & Borm, 2012; Konstantopoulos, 2012; Moerbeek, 2006; Murray & Blitstein, 2003; Raudenbush, 1997; Raudenbush, Martinez, & Spybrook, 2007), taking a pretest measurement on the response variable (Murray, 2001; Murray & Blitstein, 2003; Murray, Hannan, Wolfinger, Baker, & Dwyer, 1998; Murray, Van Horn, Hawkins, & Arthur, 2006) and taking multiple measurements at baseline and endline (Copas & Hooper, 2020).

Another strategy involves implementing a multi-period design within a cluster randomized trial such that the study becomes longitudinal. The duration of the trial is split into periods, such as days, weeks or months. Within each period a treatment is implemented within each cluster, and subjects are measured on their outcome variables. Introducing multiple periods raises questions with respect to the design and analysis of cluster randomized trials. What is the optimal trade-off between study duration, the number of periods, the number of clusters and the number of subjects per cluster per period? What is the increased efficiency of a cohort versus a repeated cross-sectional design? What is the increased efficiency of a crossover or stepped-wedge design versus a parallel-group design? What is the appropriate model for data obtained from multi-period trials, and which is the best estimation method? How do we model the correlation between outcomes of subjects within the same or different period? How does the correlation structure influence efficiency? Over the past two decades, dozens of papers on the design and analysis of multi-period cluster randomized trials have appeared, especially in the biostatistical literature (Giraudeau, Ravaud, & Donner, 2008; Grantham, Kasza, Heritier, Hemming, & Forbes, 2019; Hooper & Bourke, 2015; Rietbergen & Moerbeek, 2011).

A question that has received too little attention thus far is: what are the effects of missing data on design efficiency in multi-period cluster randomized trials? Two types of missing data can be distinguished: those that are planned by the researcher and those that are not. The former are referred to as planned missing data designs (Rhemtulla, Jia, & Little, 2014; Wu, Jia, Rhemtulla, & Little, 2016). Such designs occur when, for generally practical reasons, the researcher plans not to record all the measures that would be desirable, but only some, for instance to lower the burden on the clusters. In this case the registration is necessarily carried out intermittently. For instance, a researcher can plan a trial with a duration of a certain number of weeks where measurements are only taken on the five workdays of each week. In such a trial design, intermittently missing data occur at the two weekend days of each week. It would then be of interest to compare such a design with one in which measurements are taken on all seven days each week.

The other type of missing data occurs unintentionally when a researcher plans to measure on certain days, but for reasons beyond his or her control, measurements cannot be taken on some of these days. Such unplanned missing data can describe an intermittent or monotone pattern (or a combination of the two), depending on the position of the missing values within the longitudinal study design. Intermittently missing data, also known as non-monotone or general missing data, are missing within a trajectory: there are missing observations between the observed. Monotone missing data are missing either at the beginning or at the end of a trajectory. The monotone missing data include the case of left- or right-censored follow-ups. If measurements cannot be taken on a certain day, then all the following (respectively, preceding) days are also missing (Genolini, Écochard, & Jacqmin-Gadda, 2013). Right-censored follow-ups occur when there is dropout. Dropout is the rule rather than the exception in longitudinal research. During the course of the trial, clusters may drop out for various reasons, for instance because they were randomized to the least interesting treatment condition, because they lost interest in the trial or because they are no longer willing to put effort in recruiting, treating and measuring subjects. The effect of dropout in longitudinal trials has been studied for trials in which there is no clustering (Galbraith & Marschner, 2002; Hedeker, Gibbons, & Waternaux, 1999; Moerbeek, 2008; Molenberghs & Verbeke, 2001; Vallejo, Ato, Fernández, & Livacic-Rojas, 2019) and for cluster randomized trials (Heo, 2014; Roy, Bhaumik, Aryal, & Gibbons, 2007).

This paper extends previous research on the effect of dropout on planned missing data design efficiency of repeated cross-sectional multi-period two-arm parallel cluster randomized trials. The design is repeated in cross-sectional fashion, meaning that different sets of subjects are measured during each period and hence each subject is measured only once. The design is parallel, meaning that each cluster receives one treatment condition, and does not change treatment during the course of the trial. All calculations can be done using a Shiny app that is available on the internet. This Shiny app will be explained in further detail later in this contribution. The focus is on trials where each period is one day of the week, and where clusters are followed for either one or multiple weeks. In such trials the implementation of treatment can be done during a single day, and outcomes are measured the same day. Examples are trials that evaluate methods to reduce anxiety in the waiting room of medical care practices, trials that evaluate nutrition and hydration on the day of exams, those that evaluate a new type of equipment or procedure during surgery, and trials that evaluate distraction methods during child vaccination. There exist trials in which the period is shorter (i.e. a fraction of the day) or longer (e.g. a week or a month). The Shiny app cannot be used for such trials.

The effect of dropout is studied using the Weibull survival function to model the probability of clusters dropping out during the course of planned missing data designs, by comparing various weekly measurement schemes. This function allows for various rates of dropout and also for constant, increasing or decreasing dropout probabilities over time, the same in both groups (experimental group and control group) or different. The dropout occurs at the level of the cluster, meaning that once a cluster drops out, no further data are recorded on any subjects within that cluster. The Shiny app does not allow for dropout at the individual level.

The multilevel model is used to relate a subject’s outcome to study period and treatment condition. This model explicitly takes into account the nesting of subjects within clusters, and hence the correlation of outcomes of subjects within the same cluster. Furthermore, the correlation between outcomes of subjects within different time periods is modeled to decrease with increasing lag between these time periods. Design efficiency is measured by the variance of the treatment effect estimator. A high variance implies the design is inefficient, meaning that it has low statistical power for the test on treatment effect, whereas a low variance implies an efficient design with high statistical power. An example of a trial evaluating a method to reduce anxiety in the waiting room of dental practices is used to illustrate the findings in this paper. Throughout the paper it is assumed that the missing data mechanism is either missing completely at random (MCAR) or missing at random (MAR). In both cases the missingness is unrelated to the outcome variable. For MCAR, the missingness does not depend on other variables either, while for MAR it does depend on other variables, such as treatment condition. It is much more difficult to model informative missingness (missing not at random, MNAR), which is therefore outside the scope of this paper and not included in the Shiny app. Furthermore, the app is restricted to linear models for quantitative outcomes.

## Statistical model

**Fig. 1:**
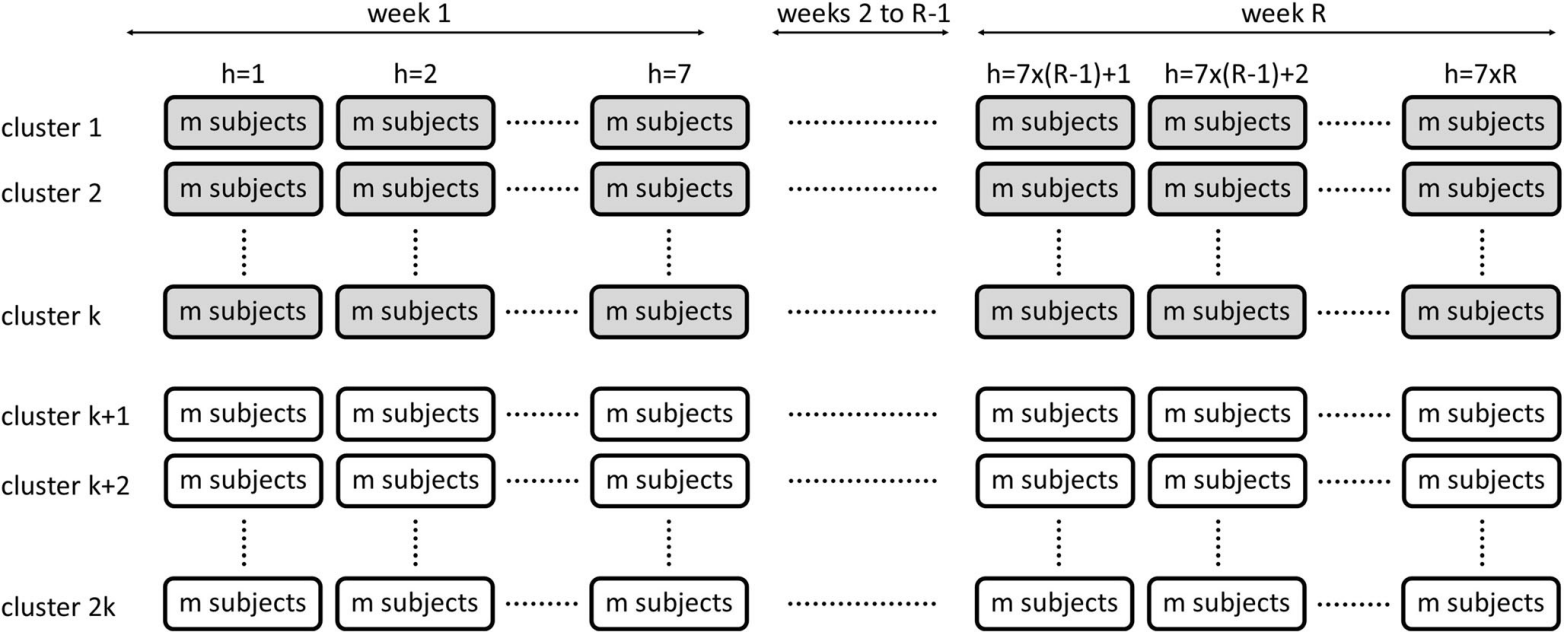
Schematic representation of the repeated cross-sectional multi-period two-arm parallel cluster randomized trial. Each box represents a cluster-period. As the design is repeated cross-sectional, different sets of subjects are included in each of the cluster-periods

This section describes the statistical model for the repeated cross-sectional multi-period two-arm parallel cluster randomized trial. A graphical presentation of this trial is given in Fig. 1. The multilevel model, also known as the mixed or hierarchical model, is used to describe the relation between treatment condition and outcome (Goldstein, 2011; Hox, Moerbeek, & Van de Schoot, 2018; Raudenbush & Bryk, 2002; Snijders & Bosker, 2012). The outcome *y*_*hij*_ of subject *i* = 1, …, *m* in period *h* = 1, …, *T* in cluster *j* = 1, …, 2*k* is given by

1 $$ {y}_{hij}={\beta}_h+{x}_j\theta +{u}_{hj}+{e}_{hij}. $$

*β*_*h*_ is the period effect for period *h*, *x*_*j*_ denotes treatment condition (0 = control, 1 = intervention), and *θ* is the effect of treatment. Note that because all period effects are included in the model, a common intercept is not required.

The model explicitly takes the hierarchical data structure into account by including a random term at the subject level, ${e}_{hij}\sim N\left(0,{\sigma}_e^2\right),$ and another one at the level of the cluster-period, ${u}_{hj}\sim N\left(0,{\sigma}_u^2\right)$. The first implies that the outcomes of subjects within a cluster-period vary across the mean score within that cluster-period. The second implies that the mean score of a cluster in a certain period varies across the mean within that period across all clusters in the same treatment condition. These two random terms are assumed to be independent from each other; hence the variance of an outcome is simply the sum of the two variance components: $\mathit{\operatorname{var}}\left({y}_{hij}\right)={\sigma}_e^2+{\sigma}_u^2$. The intraclass correlation coefficient is the proportion variance at the cluster level: $\rho ={\sigma}_u^2/\left({\sigma}_e^2+{\sigma}_u^2\right)$. It is the expected correlation between the outcomes of two randomly drawn subjects within the same cluster-period. The covariance between two outcomes from different clusters is always equal to zero, as outcomes of subjects from different clusters are assumed independent. The covariance between two outcomes within the same cluster-period is $\mathit{\operatorname{cov}}\left({y}_{hij},{y}_{h{i}^{\prime }j}\right)={\sigma}_u^2$. The covariance between two outcomes within different cluster-periods *h* and *h*^′^ is smaller and is defined as $\mathit{\operatorname{cov}}\left({y}_{hij},{y}_{h^{\prime }\ {i}^{\prime }j}\right)={\sigma}_u^2{r}^{\mid {h}^{\prime }-h\mid }$. Here we account for a non-uniform exponential decay structure: the covariance becomes smaller if the lag between the two cluster-periods increases (Grantham et al., 2019; Kasza, Hemming, Hooper, Matthews, & Forbes, 2019). This structure is also known as the first-order autoregressive structure. It should be noted that many other covariance structures are possible, such as Toeplitz, and the heterogeneous first-order autoregressive and Toeplitz structures. See for instance section 5.1 of Liu (2016) for an extensive description of such structures. Although these structures are also very common in longitudinal data research, the first-order autoregressive structure is the only structure that is applied in this contribution and its Shiny app.

The first-order autoregressive structure is the most parsimonious of these structures, as it specifies only two covariance parameters to describe all variances and covariances within a cluster across all time periods: *ρ* ∈ [0, 1] and *r* ∈ [0, 1]. As was explained above, the first is a correlation that only applies if the two subjects are within the same cluster-period. The second quantifies how much this correlation decreases if the two subjects are still in the same cluster but one period apart: (1 − *r*) × 100% is the percentage reduction in correlation. For instance, if *r* = 0.9, then the covariance decreases by (1 − *r*) × 100 %  = 10% per period. Say the correlation is *ρ* = 0.05. Then the correlation between two subjects one period apart is 90% of 0.05, or 0.045; the correlation between two subjects two periods apart is 90% of 0.045, or 0.0405, and so forth. If *r* = 1, then the covariance structure is uniform, which implies all covariances are equal, irrespective of the lag between any two cluster-periods. This structure is also known as compound symmetry. It results in higher correlations between cluster-periods than the first-order autoregressive structure, and as a result, the variance of the treatment effect estimator is higher, especially when (1 − *r*) is large (Grantham et al., 2019; Kasza et al., 2019). Therefore, the compound symmetry correlation structure results in lower power.

The model for cluster *j* in matrix notation is

2 $$ {\boldsymbol{y}}_j={\boldsymbol{X}}_j\boldsymbol{\gamma} +{\boldsymbol{Z}}_j{\boldsymbol{u}}_j+{\boldsymbol{e}}_{\boldsymbol{ij}}. $$

***y***_*j*_ is a vector of length *m* × *T* of responses in cluster *j*, and ***X***_*j*_ is the *m* × *T* by *T* + 1 design matrix for the fixed parameters in cluster *j*. ***γ =*** (*β*_1_, *β*_2_, …, *β*_*T*_, *θ*)′ is the vector of length *T* + 1 with the effects of period and treatment. ***Z***_*j*_ is the *m* × *T* by *T* design matrix for the random cluster-level effects in cluster *j*. ***e***_*ij*_ is the vector of length *m* × *T* with random subject-level effects. ${\boldsymbol{e}}_{ij}\sim {N}_{m\times T}\left(\mathbf{0},{\sigma}_e^2{\boldsymbol{I}}_j\right)$ with ***I***_*j*_ the *m* × *T* by *m* × *T* identity matrix in cluster *j*. ***u***_*j*_ = (*u*_*j*1_, *u*_*j*2_, …, *u*_*jT*_)′ is the vector of length *T* with random cluster-level effects. ${\boldsymbol{u}}_j\sim {N}_T\left(0,{\sigma}_u^2{\boldsymbol{R}}_{j,h{h}^{\prime }}\right)$ with ${\boldsymbol{R}}_{j, hh\prime }=\mathit{\operatorname{cov}}\left({y}_{hij},{y}_{h^{\prime }\ {i}^{\prime }j}\right)={\sigma}_u^2{r}^{\mid {h}^{\prime }-h\mid }$.

The covariance matrix of the outcomes in cluster *j* is calculated as

3 $$ \mathit{\operatorname{cov}}\left({\boldsymbol{y}}_j\right)={\boldsymbol{V}}_j={\sigma}_u^2{\boldsymbol{Z}}_j{\boldsymbol{R}}_j{\boldsymbol{Z}}_j^{\prime }+{\sigma}_e^2{\boldsymbol{I}}_j. $$

The regression coefficients are estimated as

4 $$ \hat{\boldsymbol{\gamma}}={\left({\sum}_{j=1}^{2k}{\boldsymbol{X}}_j^{\prime }{\hat{\boldsymbol{V}}}_j^{-1}{\boldsymbol{X}}_j\right)}^{-1}{\sum}_{j=1}^{2k}{\boldsymbol{X}}_j^{\prime }{\hat{\boldsymbol{V}}}_j^{-1}{\boldsymbol{y}}_j $$

with associated covariance matrix

5 $$ c\hat{o}v\left(\hat{\boldsymbol{\gamma}}\right)={\left({\sum}_{j=1}^{2k}{\boldsymbol{X}}_j^{\prime }{\hat{\boldsymbol{V}}}_j^{-1}{\boldsymbol{X}}_j\right)}^{-1}. $$

The element in row *T* + 1 and column *T* + 1 is the variance of the treatment effect estimator, $\mathit{\operatorname{var}}\left(\hat{\theta}\right)$, which has our primary attention in the remainder of this paper. This variance indicates how efficiently the treatment effect is estimated. A low variance is preferred, as it implies an efficient estimate and hence a high power for the test on treatment effect. High variance, on the other hand, implies an inefficient estimate and hence low power. A simple analytical expression for $\mathit{\operatorname{var}}\left(\hat{\theta}\right)$ is difficult to obtain, especially when the trial includes many cluster-periods or in the case of missing data. For that reason, $\mathit{\operatorname{var}}\left(\hat{\theta}\right)$ is calculated numerically in the software R (R Core Team, 2020), using the function solve to invert matrices. An R shiny app (Chang, Cheng, Allaire, Xie, & McPherson, 2016) is made available to calculate $\mathit{\operatorname{var}}\left(\hat{\theta}\right)$ of various designs and to compare these designs with each other. Shiny is a working environment for the development of web applications in the R language. The application is an interactive app for applied researchers and methodologists that allows easy access to a series of tools for evaluating the effect of planned missing data and dropout on design efficiency and statistical power in repeated cross-sectional multi-period two-arm parallel cluster randomized trials. The Shiny app will be described in further detail in Section 4.

## An example

Anxiety in patient-centered care is linked to negative health outcomes, such as longer recovery periods, lower pain thresholds and resistance to treatment. Various methods aimed at reducing pre-procedure waiting anxiety have been proposed: music, aromatherapy, interior design features, play opportunities and media distractions (Biddiss, Knibbe, & McPherson, 2014).

Leather, Beale, Santos, Watts, and Lee (2003) compared two different types of waiting room environments in a United Kingdom neurology outpatient waiting area, a so-called nouveau environment and a traditional environment. These environments differed with respect to various features including general layout, color scheme, floor covering and lighting. The two types of environments were compared on outcome measures such as self-reported stress and anxiety, satisfaction ratings and pulse readings. The authors concluded that the physical design of the hospital environment is an important and integral part of the therapeutic milieu.

Suppose this trial is to be replicated in another setting in another country, say in dentistry care in the Netherlands. It is obvious that multiple dental practices are to be recruited so that a sufficient number of patients can be enrolled in a limited amount of time, and so that the results will be generalizable to all dental practices in the country. The redesign of the waiting areas may be expensive, so a trial like this will obviously run for multiple weeks to justify these trial costs. In theory, a crossover or stepped-wedge trial design would be possible. The advantage of clusters changing their treatment during the course of a trial is increased efficiency. However, in this specific trial this advantage may be outweighed by increased costs due to the redesign of the waiting areas during each crossover. Also, redesigning multiple waiting areas during the course of a weekend may be difficult to achieve, and it may also result in increased dropout rates. The parallel-group design is therefore the most obvious choice.

## Shiny app

The Shiny app is available online at https://utrecht-university.shinyapps.io/missing_data_CRT/. It can be used to plan a repeated cross-sectional multi-period two-arm parallel cluster randomized trial and in four different situations:

1. When there is no data loss. In this case, data are recorded for seven days a week for at least one week, and dropout is absent. Section 5 shows how design efficiency is influenced by the number of weeks and number of subjects per cluster-period, for various values of *ρ* and *r*.
2. Trials where the registration is carried out during a certain number of days of the week. In other words, the trial is planned under the planned missing data design umbrella, and there is no data loss due to dropout. Section 6 considers four conditions of a planned missing data design with measurements on five or fewer days of the five workdays (Monday through Friday) and compares these to the complete data design (i.e. the design from situation 1).
3. Trials for which measurements are taken seven days of the week and where dropout occurs. In Section 7, the Weibull survival function is used to describe the amount of dropout and whether the risk of dropout increases, decreases or remains constant during the course of the trial. Designs with various dropout patterns will be compared with the complete data design.
4. Trials with intermittently planned missing observations with dropout. In Section 8 the example from the previous section is revisited. Measurements are taken Mondays through Fridays, and designs with either 4 or 8 weeks and with either 10 or 15 clusters per condition are compared in a situation where dropout occurs.

The Shiny app allows for the specification of five designs. Whenever acceptable values have been entered, it returns the results for those five designs. The user has to pay attention to the designs that are most convenient in his/her trial by changing the default input. The top part of the Shiny app allows the user to specify the designs, the correlation parameters, details about the statistical test on treatment effect, and the dropout pattern in both experimental conditions. The right part of the bottom shows the variance of the treatment effect estimator, power and design efficiency in graphical and table format.

The left part at the top allows the user to specify the design parameters. The range of the number of subjects per day is specified at the top. Below that, five different designs can be specified by selecting the days of the week in which measurements are taken, along with the number of weeks (*R* ≥ 1) and number of clusters per condition (*k* ≥ 1). It should be noted that designs should be specified such that measurements are taken on at least two days. So a design with one week and one day will generate a warning. Note that the five default sets of days of the week are those that are used in Section 6. However, the user can select any number and combination of days of the week. In the middle part at the top, the correlation parameters *ρ* ∈ [0, 1] and *r* ∈ [0, 1] should be specified, along with the details for the test on treatment effect (effect size Cohen’s *d* ≠ 0, type I error rate *α* ∈ [0, 1] and whether the test is one- or two-sided). In the right part at the top, the parameters of the Weibull survival function should be specified (*ω* ∈ [0, 1] and *γ* > 0), along with the maximum duration of the trial (*t*_*max*_ ≥ 1). These parameters will be explained in further detail in Section 7; for now it is important to understand that the parameter *ω* should be equal to zero in the case where there is no dropout (situations 1 and 2) and between 0 and 1 if there is dropout (situations 3 and 4). The app allows different dropout patterns for the two experimental conditions. The hazard and survival probability functions are displayed in the second and third tabs. The user may consult Table 11.1 in Moerbeek and Teerenstra (2016) for a priori estimates of *ρ*. Unfortunately, such an extensive overview of estimates does not exist for *r*, *ω* or *γ*, and the user is encouraged to search the literature for similar studies, or use experts’ opinions or expectations.

Once all input has been specified, the submit button at the bottom left should be pressed. Calculating the output may take a while, especially when the number of subjects per cluster-period is large; the progress is shown at the bottom right. The output is given in three graphs (variance of the treatment effect estimator, power for the test on treatment effect, and efficiency of designs 2-–5 as compared to design 1). The same output is given in table format; the number of subjects per cluster-period is shown in the first column, the variance of the treatment effect estimator of designs 1-–5 shown in the next five columns, the power of these designs in the next five columns, and their efficiencies as compared to design 1 in the last five columns.

## Trials without missing data

This section summarizes how the variance of the treatment effect estimator behaves in trials without missing data. For more extensive results the reader is referred to related papers in the medical statistical literature (Grantham et al., 2019; Kasza et al., 2019). The variance depends on the intraclass coefficient *ρ* and decay parameter 1 − *r* and on the design *ξ* = (*k*, *m*, *T*), where *k* is the number of clusters per condition, *m* is the number of subjects per cluster-period and *T* is the number of periods. The design can also be written as *ξ* = (*k*, *m*, *R*), where *R* is the number of consecutive weeks over which measurements are taken. In the case where measurements are taken on each of the seven days of the week, the number of periods is *T* = 7 × *R*.

**Fig. 2:**
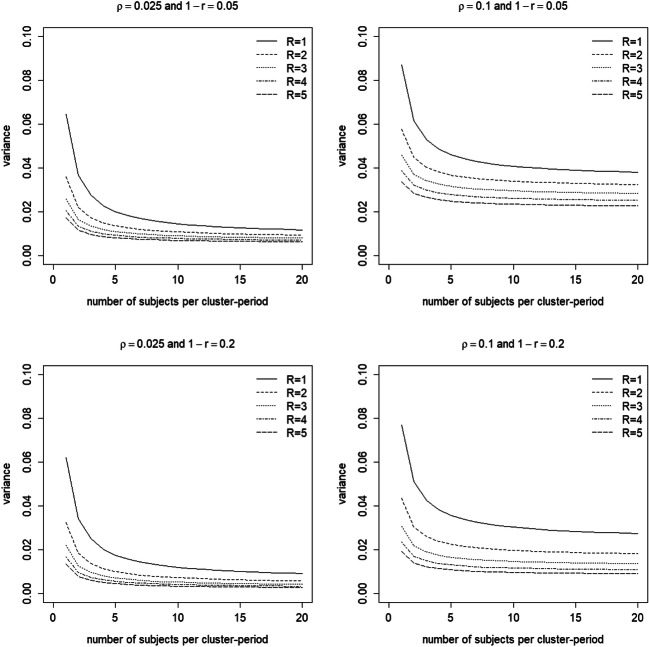
Variance of the treatment effect estimator as a function of the number of measurements per cluster-period (*m*, horizontal axis within each graph), number of weeks (*R*, lines within each graph) and for four combinations of the intraclass correlation coefficient *ρ* and decay parameter 1 − *r* (separate graphs). The number of clusters per condition is *k* = 5

Figure 2 shows the variance of the treatment effect estimator as a function of the number of subjects per cluster-period *m*, number of weeks *R*, the intraclass correlation coefficient *ρ* and decay parameter 1 − *r*. The variances are calculated for trials with *k* = 5 clusters per treatment condition. As explained previously, the variance is a measure of efficiency: the higher the variance, the less efficient the design.

Figure 2 shows that the variance increases with increasing intraclass correlation *ρ*. This is not surprising, as the same relation holds for cluster randomized trials with just one period: the higher the intraclass correlation, the higher the correlations between the outcomes of subjects in the same cluster, the less information there is in the data, and hence the higher the variance of the treatment effect estimator. Figure 2 also shows that the variance decreases with increasing decay 1 − *r*. A higher decay implies a lower correlation between outcomes in the same cluster, and hence a lower variance, but this parameter is most often not under the control of the researcher. By using the Shiny app, the reader can easily verify that highest variance is obtained with the compound symmetry correlation structure, for which 1 − *r* = 0.

Figure 2 also shows that the variance decreases with increasing number of subjects per cluster-period *m*, especially when the number of weeks *R* is low. However, this effect becomes negligible when the number of subjects per cluster-period becomes larger than 5, and the variance approaches a limit when the number of subjects per cluster-period further increases. A similar relation holds for cluster randomized trials with just one period: increasing the cluster size has some effect when cluster size is small, but the effect becomes minimal at larger cluster sizes (Hemming, Girling, Sitch, Marsh, & Lilford, 2011; Moerbeek & Teerenstra, 2016; Raudenbush, 1997). Furthermore, Fig. 2 shows that the variance decreases with increasing number of weeks, especially when the number of weeks is small. This is also obvious, since a larger study duration implies that more measurements are taken and hence a lower variance is achieved.

As follows from Eq. (5), the variance is inversely related to the number of clusters, a relation that also holds for cluster randomized trials with one period (Moerbeek & Teerenstra, 2016; Raudenbush, 1997). For instance, doubling the number of clusters results in a variance that is half as large. Changing the number of clusters implies rescaling the vertical axis in Fig. 2, while the effects of the other two design factors (*m* and *R*) remain unchanged for any given *ρ* and 1 − *r*. For that reason, other values of the number of clusters are not considered in this figure or the figures in the next two sections.

## Trials with intermittently planned missing observations without dropout

The results in the previous session are based on trials in which measurements can be taken on all seven days of the week. This may indeed be the case in, for example, a trial with patients nested within hospitals where two different methods to relieve stress and anxiety in the emergency room are compared. In other trials, however, the clusters may be health professionals such as psychotherapists or dentists, who most often do not work seven days a week. The aim of this section is to compare planned missing data designs with fewer than seven days of the week to the design with seven days of the week. As we do not consider dropout, the parameter *ω* should be fixed to zero in the Shiny app for both conditions.

The following five measurement schemes are taken into account:

- $$ {S}_1=\left\{ Mo, Tu, We, Th, Fr, Sa, Su\right) $$
- $$ {S}_2=\left\{ Mo, Tu, We, Th, Fr\right) $$
- $$ {S}_3=\left\{ Mo, Tu, Th, Fr\right) $$
- $$ {S}_4=\left\{ Mo, Tu, We, Th\right) $$
- $$ {S}_5=\left\{ Mo, Tu, Th\right) $$

With scheme *S*_1_, measurements are taken on all seven days, and with scheme *S*_2_, measurements are taken on the five workdays only. With the other three schemes, measurements are taken on either three or four days of the workweek. These schemes are rather typical in the Netherlands, where many elementary schools are closed on Wednesday and/or Friday afternoons and during the two days of the weekend. Parents who have elementary school children and do not work full-time most often have a day off each Wednesday or Friday or even both.

The efficiency of measurement scheme *S*_*s*_ relative to scheme *S*_*t*_ is calculated as

6 $$ RE=\frac{\mathit{\operatorname{var}}{\left(\hat{\theta}\right)}_{S_t}}{\mathit{\operatorname{var}}{\left(\hat{\theta}\right)}_{S_s}}, $$

where the numerator and denominator are the variance of the treatment effect estimator obtained with schemes *S*_*t*_ and *S*_*s*_, and *s*, *t* = 1, 2, 3, 4, 5, respectively. The inverse of the relative efficiency indicates how often measurement scheme *S*_*s*_ should be replicated to do as well as *S*_*t*_. In most practical situations, relative efficiencies above 0.8 or 0.9 are favored. *RE* = 0.9 implies that a trial with scheme *S*_*s*_ should include $\left(\frac{1}{0.9}-1\right)\times 100\%=11\%$ extra clusters to do as well as a trial with scheme *S*_*t*_; for *RE* = 0.8 an increase of 25% is needed.

**Fig. 3:**
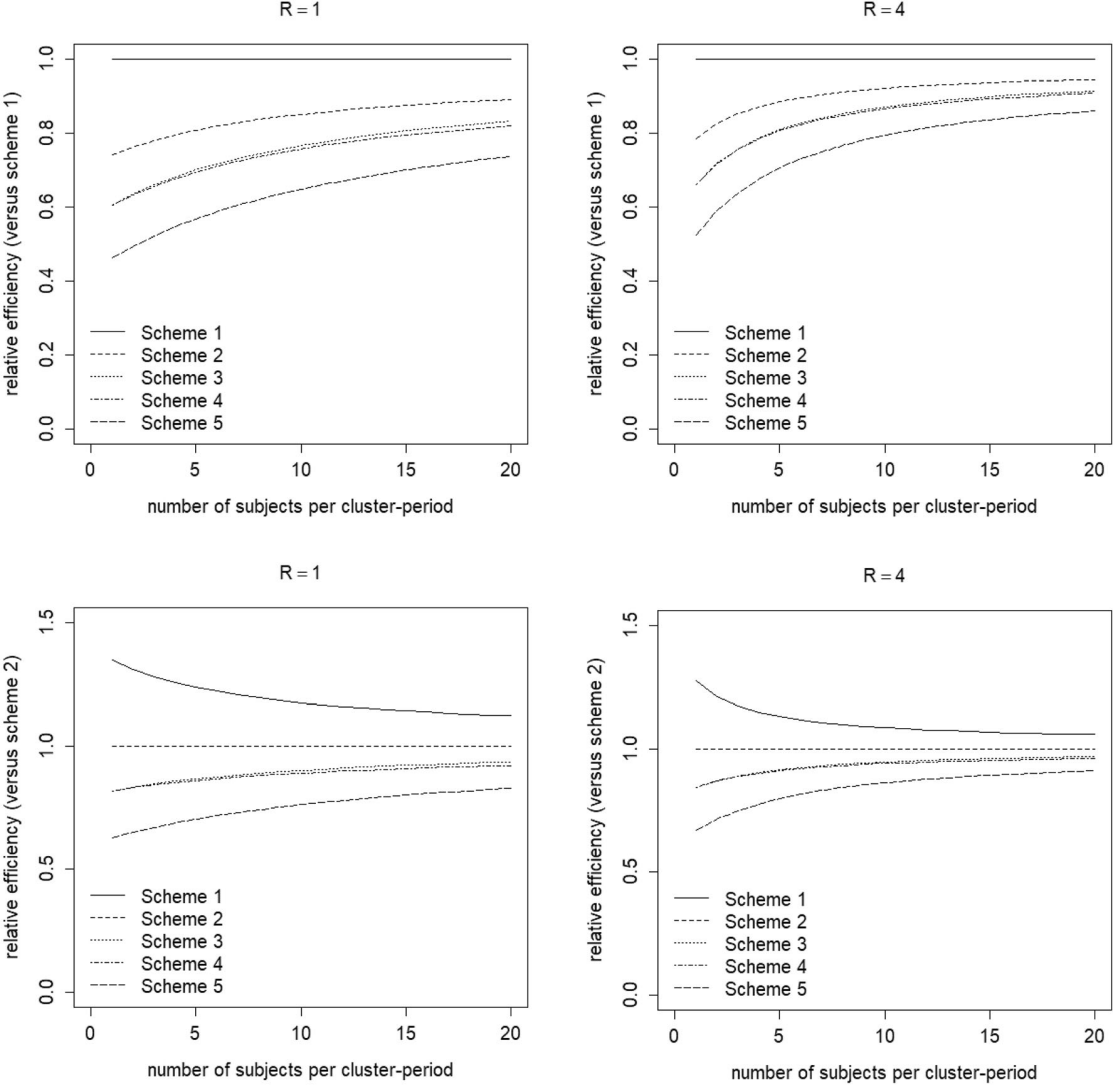
Efficiency of the treatment effect estimator for various measurement schemes (separate lines) as compared to scheme 1 (top panels) and scheme 2 (bottom panels), as a function of the number of measurements per cluster-period *m* (horizontal axis) and the number of weeks *R* (separate panels). Intraclass correlation coefficient *ρ* = 0.025 and decay parameter 1 − *r* = 0.05

The two top panels of Fig. 3 show the efficiency of measurements schemes *S*_2_–*S*_5_ relative to measurement scheme *S*_1_ as a function of the number of subjects per cluster-period *m* and for two different values of the number of weeks *R*. This figure holds for (*ρ*, 1 − *r*) = (0.025,0.05). As is obvious, lower efficiency is achieved when measurements are taken on fewer days. The relative efficiencies of measurement schemes *S*_3_ and *S*_4_ are almost the same, since both include four days. The lag between the first and the last day is larger for scheme *S*_3_ than for *S*_4_, and hence it has a slightly larger relative efficiency. A similar finding holds for longitudinal intervention studies with repeated measurements within subjects (Moerbeek, 2008): higher efficiency is achieved when study duration increases (while keeping the number of days constant). The loss in efficiency may be considerable: in most scenarios considered in Fig. 3 it is below 0.9, and in extreme cases it may be as low as 0.47.

For any measurement scheme *S*_2_ through *S*_5_, the loss of efficiency is larger for small numbers of subjects per cluster-period *m* and for small numbers of weeks *R*. This is unfortunate since, as was shown in Fig. 2, the highest variance is achieved with the lowest number of subjects per cluster-period and lowest number of weeks. In other words, the combination (*m*, *R*) with highest variance suffers the most from not measuring on all seven days of the week.

In the two top panels of Fig. 3, the scheme with measurements on all seven days is used as reference for calculating the relative efficiency. It is of course possible to use any other measurement scheme as reference, for instance the scheme with measurements on the five workdays (scheme *S*_2_). The results of this comparison are presented in the two bottom panels of Fig. 3. The schemes with four days (*S*_3_ and *S*_4_) have relative efficiencies above 0.8, so taking measurements on four rather than five workdays of the week requires a slight increase in the number of clusters. As is obvious, the measurement scheme with just three days performs worst; the relative efficiency can be as low as 0.65.

Figures S3a–S3d in the online supplement present results for four different combinations (*ρ*, 1 − *r*). For a given decay, the loss in efficiency is slightly higher if the intraclass correlation is lower. Furthermore, for a given intraclass correlation, the loss in efficiency is slightly higher if the decay is higher. As we saw in the previous section (Fig. 2), the largest variance of the treatment effect estimator was observed for high intraclass correlation and low decay. Thus the combination (*ρ*, 1 − *r*) with the highest variance suffers the least from measuring on fewer days of the week. The figures in the supplement also show that for the other combinations (*ρ*, 1 − *r*), the relative efficiencies of schemes *S*_3_ and *S*_4_ differ somewhat more than for (*ρ*, 1 − *r*) = (0.025,0.05).

In many cases the health professionals that are included will not work on exactly the same days, meaning that a trial uses a mixture of various measurement schemes: *S*_*s*_ = ∑_*l*_*w*_*l*_*S*_*l*_, with *w*_*l*_ the weight for scheme *S*_*l*_ such that 0 < *w*_*l*_ < 1 and ∑_*l*_*w*_*l*_ = 1. The variance of the treatment effect estimator is calculated as explained in section 2, and plugged into Eq. (6) to calculate the relative efficiency.

## Trials with dropout

Dropout occurs when clusters drop out of the study and do not return in later periods. Dropout may vary across the two experimental conditions. It may be higher in the control condition in the case where clusters in this condition are less motivated because they were not randomized to a new and promising intervention and are therefore less willing to recruit patients for a long amount of time. On the other hand, dropout may be higher in the intervention condition if the intervention puts a large burden on the clusters and subjects or when it has harmful side effects.

It is assumed that dropout can occur on any day, not necessarily at the end of a week. For the sake of simplicity, we assume dropout only occurs at the end of a day, not during a day. This implies that dropout is discrete rather than continuous. The effect of dropout depends on how many clusters drop out and when they do so. We use basic concepts of discrete-time survival analysis to model dropout patterns (Singer & Willett, 1993, 2003). The survival probability function gives a cluster *j*’s probability of staying in the trial up to at least day *t*:

7 $$ S\left({t}_{jt}\right)=P\left({T}_j\ge t\right). $$

The discrete random variable *T*_*j*_ measures the elapsed study time (i.e. number of days). The associated hazard probability function gives the probability of cluster *j* experiencing the event on day *t*, conditional on not having experienced the event up till then

8 $$ h\left({t}_{jt}\right)=P\left({T}_j=t\right|{T}_j\ge t\Big). $$

It is calculated as

9 $$ h\left({t}_{jt}\right)=\frac{S\left({t}_{j\left(t-1\right)}\right)-S\left({t}_{jt}\right)}{S\left({t}_{j\left(t-1\right)}\right)}. $$

To calculate the effect of dropout, the vector *K* = (*k*_1_, *k*_2_, …, *k*_*T*_)′, with *k*_*h*_ being the number of clusters with exactly *h* measurements, needs to be known in both treatment conditions. This vector is random; the associated probability vector is *p* = (*p*_1_, *p*_2_, …, *p*_*T*_)′. A cluster’s probability *p*_*h*_ of having exactly *h* measurements is calculated from the survival function: *p*_*h*_ = *S*(*t*_*h*_) − *S*(*t*_*h* + 1_) for *h* = 1, …, *T* − 1 and *p*_*T*_ = *S*(*t*_*T*_). This is the probability of dropping out between *t*_*h*_ and *t*_*h* − 1_. For each possible vector *K*, a probability can be calculated, and the variance of the treatment effect estimator can be calculated from (5). The expected variance is then the sum of probability × variance over all possible vectors *K*. This procedure is hardly useful in settings where the number of periods, and hence the number of vectors *K*, is large. The variance of the treatment effect can be approximated by using a sampling procedure (Verbeke & Lesaffre, 1999). The vector *K* is sampled a large number of times from the multinomial distribution with probability vector *p*. For each draw, the variance of the treatment effect estimator is calculated and the mean of the variance across all draws is used to calculate the effect of dropout. The drawback of this procedure is that it may be time-consuming, especially if the number of draws is large. A yet further approximation is made by not using a sampling procedure, but replacing the vector *K* by its expectation E(*K*) = *k* × *p*. This procedure has been compared to the sampling procedure; both produced very similar results (Galbraith & Marschner, 2002).

Many different survival functions exist; we use the Weibull survival function (Galbraith & Marschner, 2002; Moerbeek, 2008), which is given by

10 $$ S\left({t}_{jt}\right)=\exp \left(-\lambda {t}^{\gamma}\right). $$

Time is rescaled by dividing by *t*_*max*_, which is the maximum duration the trial can take. *t*_*max*_ may be based on financial or practical considerations, such as the maximal duration most clusters are willing to participate in the trial, but it may also be set by the trial’s funding organization. The parameter *λ* is replaced by − log(1 − *ω*), where *ω* ∈ [0, 1] is the proportion of clusters that drop out at some time during the course of a trial with *t*_*max*_. The survival function then becomes

11 $$ S\left({t}_{jt}\right)={\left(1-\omega \right)}^{t^{\gamma }}. $$

**Fig. 4:**
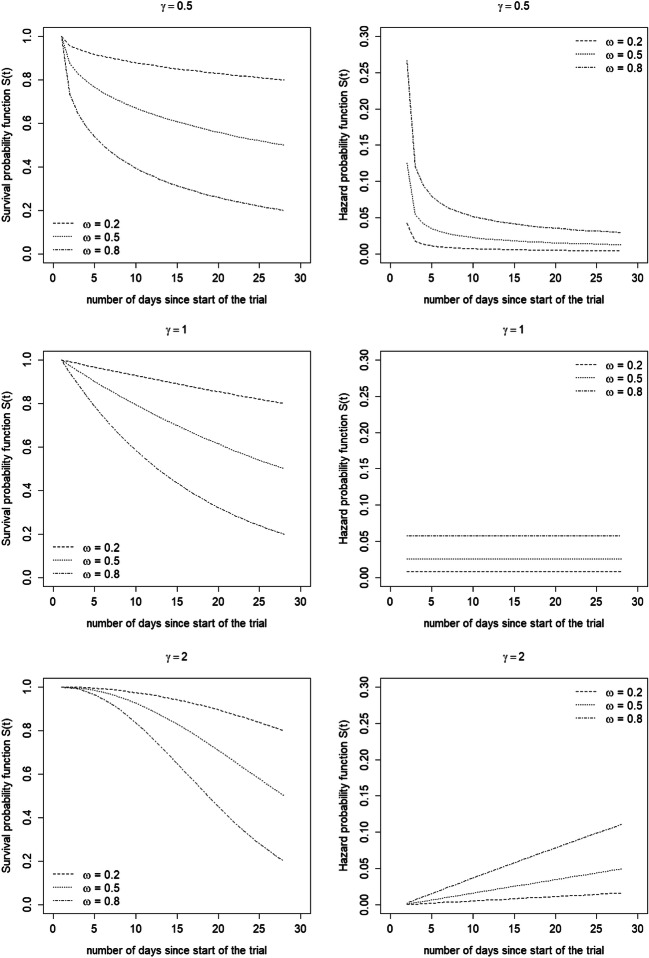
Survival and hazard probability functions for *ω*= 0.2, 0.5 and 0.8 (separate lines within each graph) and for *γ*= 1/2 (top panel), 1 (middle panel) and 2 (bottom panel)

Figure 4 shows the survival and hazard probability functions for a trial with a duration of 28 days (i.e. measurement scheme *S*_1_ and *R* = 4 weeks) for various values *ω* and *γ* and with equal dropout across the two experimental conditions. When *γ* < 1 the hazard probability decreases during the course of the study, when *γ* = 1 it is constant and when *γ* > 1 it increases. As is obvious, the hazard probability becomes larger and the survival probability becomes smaller when *ω* becomes larger.

**Fig. 5:**
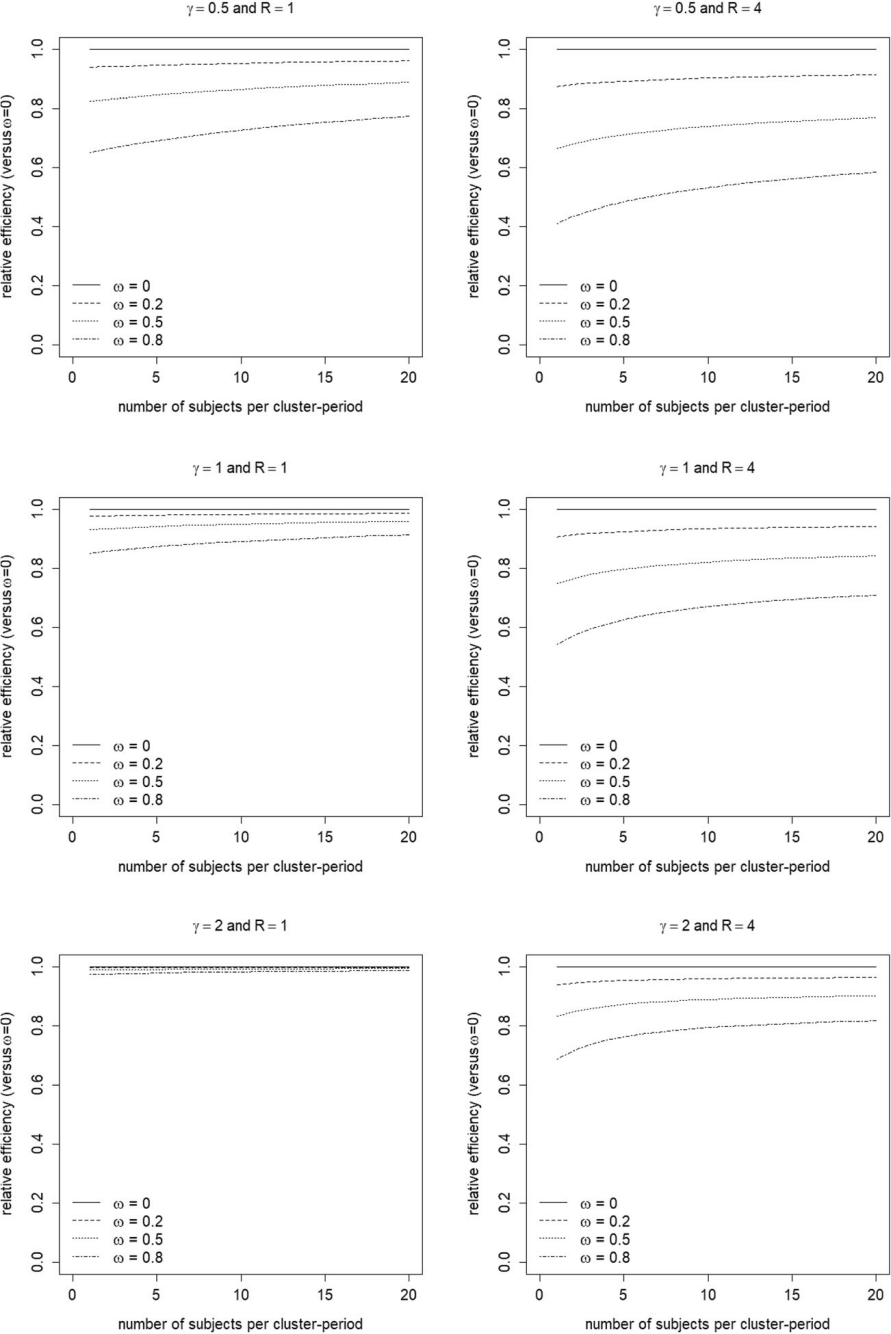
Efficiency of the treatment effect estimator for various dropout patterns (*ω*, separate lines and *γ*, separate panels) as compared to no dropout, as a function of the number of measurements per cluster-period *m* (horizontal axis) and the number of weeks *R* (separate panels). Intraclass correlation coefficient *ρ* = 0.025 and decay parameter 1 − *r* = 0.05

Figure 5 shows the effect of dropout for a trial with measurement scheme *S*_1_ as a function of the number of subjects per cluster-period *m* and for two different numbers of weeks *R*. The efficiency is displayed relative to a trial without dropout (*ω* = 0). Three values *ω* and three values *γ* are considered. Note that *ω* is the proportion dropout in a trial with a maximal duration *t*_*max*_ = 28 days (i.e. *R* = 4), as displayed in Fig. 4. The results in Fig. 5 hold for (*ρ*, 1 − *r*) = (0.025,0.05).

For each *γ*, the loss in efficiency is larger when *ω* is larger. This is obvious, since the greater the number of clusters that drop out, the larger the variance of the treatment effect estimator and hence the lower the efficiency of the trial. Furthermore, for large *γ*, the effect of *ω* is smaller than for small *γ*, especially for small *R*. This finding can be explained as follows. The survival probability function hardly varies across the three values of *ω* in a trial with just seven days (*R* = 1) and *γ* = 2, see the panel at the bottom left in Fig. 4. Hence, the relative efficiency hardly depends on the value of *ω*. The smaller the *γ*, the more the survival probability functions of the three values *ω* differ in a trial with seven days, and hence the larger the effect of *ω* on the relative efficiency. Increasing study duration implies that the survival functions for various values *ω* differ even more. Hence, the effect of *ω* on the relative efficiency becomes stronger.

Figure 5 also shows that, for each *ω*, the efficiency is larger when *γ* is smaller. This is also obvious, since a smaller *γ* implies that the hazard probability is largest at the beginning of the trial. In other words, smaller *γ* implies that there are more clusters with fewer days, so the variance becomes larger and the trial becomes less efficient.

The loss in efficiency is largest with few subjects per cluster-period *m*. This implies largest loss in efficiency for those trials that already have a large variance. Furthermore, the effect of *m* becomes smaller for large values of *m*.

Finally, a larger loss in efficiency is observed for a larger number of weeks *R*. A larger *R* implies a longer trial duration and hence a larger amount of dropout during the course of the trial. For instance, consider a trial with 28 days (*R* = 4) where half of the clusters have dropped out by the end of the trial (*ω* = 0.5). Reducing study duration to a quarter (*R* = 1) would result in a dropout proportion of 0.28 (*γ* = 1/2), 0.14 (*γ* = 1) or 0.03 (*γ* = 2). So a shorter study duration implies that fewer clusters drop out, hence a smaller loss of efficiency. Therefore, studies that have higher variance of the treatment effect estimator due to a short study duration suffer the least from dropout.

The Online Supplement shows relative efficiencies for four combinations of the intraclass correlation coefficient *ρ* and decay parameters 1 − *r*, see Figs. S5a–S5d. The effects of *ρ* and 1 − *r* are as in the previous section: for a given decay 1 − *r*, the loss in efficiency is slightly larger for a smaller *ρ*, and for a given *ρ* the loss in efficiency is slightly larger for a larger 1 − *r*. Thus the combination (*ρ*, 1 − *r*) with the highest variance suffers the least from dropout.

## Trials with intermittently planned missing observations with dropout

Here we revisit the example in Section 3 to demonstrate the evaluation of designs of trials with intermittently planned missing observations with dropout. The data should become available within at most 8 weeks. It is likely that some dental practices may drop out. Let us suppose that the dropout rate is *ω* = 0.2 in traditional waiting areas and *ω* = 0.1 in nouveau waiting areas. Dropout may be expected to increase slightly during the course of the trial as dental practices lose motivation to ask their patients to participate. Such a dropout pattern is achieved by choosing *γ* > 1; let us assume that *γ* = 2. To calculate the efficiency of the design, a priori estimates of the intraclass correlation coefficient and decay parameter must be specified. Let us assume that *ρ* = 0.05 and 1 − *r* = 0.05.

In the Netherlands, dental practices are typically closed during the weekend. We compare four designs with a different number of dental practices per condition and different numbers of weeks. All designs include all five workdays:

- $$ Design\ 1:k=10,R=4, Scheme=\left( Mo, Tu, We, Th, Fr\right) $$
- $$ Design\ 2:k=15,R=4, Scheme=\left( Mo, Tu, We, Th, Fr\right) $$
- $$ Design\ 3:k=10,R=8, Scheme=\left( Mo, Tu, We, Th, Fr\right) $$
- $$ Design\ 4:k=15,R=8, Scheme=\left( Mo, Tu, We, Th, Fr\right) $$

The study should be designed so that it has a power of at least 80% in a two-sided test with a type I error rate *α* = 0.05. Leather et al. (2003) found a zero effect for patients’ rating on anxiety, and small effects for disability (Cohen’s *d* = 0.22) and pain (Cohens *d* = 0.27). In the calculations that follow we assume a small effect size Cohen’s *d* = 0.2.

**Fig. 6:**
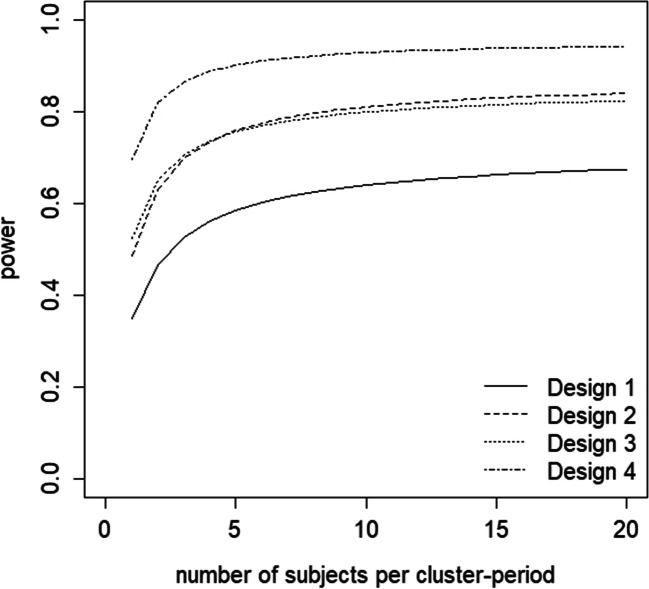
Power levels as a function of the number of subjects for the four designs in the waiting room example

Figure 6 shows the power as a function of the number of patients per day. Obviously, higher power is achieved when more patients are observed per day, but for all designs, the power levels off to a certain limit when the number of patients per day increases. The lowest power is observed for design 1. With this design, sufficient power cannot be achieved even when as many as 20 patients per day are included. Designs 2 and 3 have comparable power levels. Design 3 has slightly higher power when at most four patients per day are included, while design 2 has slightly higher power for at least five patients per day. The number of patients to be included per day to achieve at least 80% power is 9 and 11 for designs 2 and 3, respectively. The largest power is achieved with design 4: only two patients per day are needed to achieve at least 80% power.

Based on some criterion, the best design can be chosen from designs 2-4. If, for example, trial duration is to be minimized, then design 2 is the best choice. If, as another example, recruiting dental practices is difficult, then design 3 should be chosen. If, as yet another example, the total number of patients should be minimized, then design 4 should be chosen.

The impact of using a measurement scheme with fewer days may also be studied. For *Scheme* = (*Mo*, *Tu*, *Th*, *Fr*), the required number of patients per day is 11 (design 2), 13 (design 3) and 3 (design 4). For *Scheme* = (*Mo*, *Tu*, *Th*), the number of patients per day is 15 (design 2), 18 (design 3) and 3 (design 4). Therefore, including fewer days in the design results in a larger number of subjects to be measured per day to achieve the desired power level.

## Conclusions and discussion

Missing data result in a loss of efficiency. In the case of planned missing data designs, measurements are not taken on certain days of the week. The fewer the number of days in which measurements are taken, the lower the efficiency. In addition, lower efficiency is obtained if the lag between the first and last day of the week in which measurements are taken becomes smaller. Furthermore, the loss in efficiency in planned missing data designs is largest for small numbers of subjects per period and small numbers of weeks. In the case of unplanned missing data resulting from dropout, clusters drop out of the trial and do not return in later periods. The higher the amount of dropout, the larger the loss of efficiency, especially if dropout is largest at the beginning of the trial. The loss in efficiency due to dropout is largest for small numbers of subjects per period and large numbers of weeks. In both planned and unplanned missing data designs, the loss of efficiency increases when the size of the intraclass correlation decreases and the decay parameter increases.

The relation between sample size, number of weeks and missing data patterns, on the one side, and variance of the treatment effect estimator, efficiency and power on the other side cannot be captured by a simple mathematical relationship. For this reason, it may be explored by using the Shiny app. Various designs may be specified and the power for each of them can be calculated. Among all designs with sufficient power, the best one may be chosen based on some criterion, such as shortest study duration, smallest number of clusters or smallest number of measurements.

To use the Shiny app, the values of the intraclass correlation coefficient and decay parameter should be specified. The values of such parameters are often unknown in the design phase of a trial. An a priori guess may be based on researchers’ expectations, expert opinion or findings in the literature. Over the past two decades, estimates of intraclass correlation coefficients in cluster randomized trials with one period have been published; see Table 11.1 of Moerbeek and Teerenstra (2016) for an overview of such papers. It is important that such estimates also become available for multi-period cluster randomized trials, along with estimates of the decay parameter. Furthermore, it would be very helpful if the dropout that occurred within a trial would be described in detail to facilitate planning future trials. Not only should the total amount of dropout during the course of a trial be given, but the hazard probability function should be given as well. Finally, the size of the effect must be specified. When a priori estimates of Cohen’s *d*, *ρ*, 1 − *r*, *ω* and *γ* cannot be found in the literature, then the researcher may be able to come up with a range of plausible values of these parameters. Then, the worst-case scenario (i.e. smallest *d*, largest *ρ*, smallest 1 − *r*, largest *ω* and smallest *γ*) may be used in the Shiny app.

In this paper the Weibull function was used to model the probability of dropout in each of the time periods. This is a very flexible function, as it allows for constant, increasing or decreasing hazard over time. Of course, there exist many other survival functions, and it would even be possible to let the researcher specify the hazard probability in each of the time periods. However, this is outside the scope of this paper. Furthermore, the probability of dropout was assumed to depend on treatment condition and time elapsed since the start of the study. There may be other factors that influence dropout, for instance the number of days per week in which measurements are taken or the number of subjects who have to be measured per day. It would be interesting to explore more complicated survival and hazard functions in future research.

This paper is restricted to a repeated cross-sectional design, meaning that each subject is measured only once. Higher efficiency is achieved by using a cohort design. For such a design, the statistical model needs to be extended to accommodate repeated measures within subjects. Sample size calculations for such a model are given in Hooper, Teerenstra, De Hoop, and Eldridge (2016), but that study is restricted to a compound symmetry structure and does not take missing data into account. Future research should focus on exponential decay at both the cluster and subject level. It is also possible to include multiple cohorts, and this may be done in a serial or parallel manner. In the first case, cohorts are observed one after the other, while measurements across multiple days are taken within each cohort. In the second case, subjects are measured on a fixed day of the week during the course of multiple weeks. Furthermore, it is also possible to implement a combination of a cohort and repeated cross-sectional design.

The focus of this paper is on a parallel-arm design, meaning that all subjects within the same cluster receive the same treatment. Such a design is often chosen if there is a risk of contamination of the control condition (Moerbeek, 2005). Such contamination is likely to occur in trials where the cluster is a therapist. Although in theory it would be possible to let each therapist offer the intervention and control, in practice it would be difficult for therapists not to let clients in the control condition benefit from the intervention. The parallel-arm design may also be preferred for financial or practical reasons. In the example in the previous section, it would not be cost-effective to redesign the waiting area during the course of the trial in all dental practices. If such objections do not exist, a design in which clusters are exposed to both treatment conditions may be chosen to increase efficiency. With a crossover trial, clusters cross back and forth between the control and intervention conditions. In the example in the previous section, this might have been possible if the trial evaluated the effect of music or aromatherapy. The effect of dropout in a two-period crossover cluster randomized trial has been studied previously (Moerbeek, 2020), and an extension should be made to designs with more than one crossover. Another type of trial in which all clusters receive both treatment conditions is the stepped-wedge design. Here all clusters start in the control, and there is a sequential rollout of the intervention across all clusters. Kasza and Forbes (2019) studied the information content in each of the cluster-periods and found that the most information-rich are those cluster-periods that occur immediately before and after the switches. It would also be interesting to study the effect of dropout in stepped-wedge cluster randomized trials.

In summary, this paper presents new results concerning the effect of dropout on planned missing data design efficiency in repeated cross-sectional multi-period two-arm parallel cluster randomized trials. The Shiny app enables researchers to evaluate their design with respect to power and to compare its efficiency with competing designs.

## Supplementary Information

ESM 1 (DOCX 269 kb)
