# Supplementary material for: The effect of missing data on design efficiency in repeated cross-sectional multi-period two-arm parallel cluster randomized trials
Source: Behav Res Methods. 2021 Feb 2;53(4):1731–45. doi: 10.3758/s13428-020-01529-7 (PMC8367915; doi:10.3758/s13428-020-01529-7)
Supplement: Supplementary file 1 — (DOCX 269 kb) [file 13428_2020_1529_MOESM1_ESM.docx]

Figure S3a. Efficiency of the treatment effect estimator for various measurement schemes (separate lines) as compared to scheme 1 (top panels) and scheme 2 (bottom panels), as a function of the number of measurements per cluster-period $m$ (horizontal axis) and the number of replications $R$ (separate panels).

Intraclass correlation coefficient $\rho=0.025$ and decay parameter $1-r=0.05$.

Figure S3b. Efficiency of the treatment effect estimator for various measurement schemes (separate lines) as compared to scheme 1 (top panels) and scheme 2 (bottom panels), as a function of the number of measurements per cluster-period $m$ (horizontal axis) and the number of replications $R$ (separate panels).

Intraclass correlation coefficient $\rho=0.1$ and decay parameter $1-r=0.05$.

Figure S3c. Efficiency of the treatment effect estimator for various measurement schemes (separate lines) as compared to scheme 1 (top panels) and scheme 2 (bottom panels), as a function of the number of measurements per cluster-period $m$ (horizontal axis) and the number of replications $R$ (separate panels).

Intraclass correlation coefficient $\rho=0.025$ and decay parameter $1-r=0.2$.

Figure S3d. Efficiency of the treatment effect estimator for various measurement schemes (separate lines) as compared to scheme 1 (top panels) and scheme 2 (bottom panels), as a function of the number of measurements per cluster-period $m$ (horizontal axis) and the number of replications $R$ (separate panels).

Intraclass correlation coefficient $\rho=0.1$ and decay parameter $1-r=0.2$.

Figure S5a. Intraclass correlation coefficient $\rho=0.025$ and decay parameter $1-r=0.05$.

Figure S5b. Intraclass correlation coefficient $\rho=0.1$ and decay parameter $1-r=0.05$.

Figure S5c. Intraclass correlation coefficient $\rho=0.025$ and decay parameter $1-r=0.2$.

Figure S5d. Intraclass correlation coefficient $\rho=0.1$ and decay parameter $1-r=0.2$.
